# Spatiotemporal Winter Wheat Water Status Assessment Improvement Using a Water Deficit Index Derived from an Unmanned Aerial System in the North China Plain

**DOI:** 10.3390/s23041903

**Published:** 2023-02-08

**Authors:** Vita Antoniuk, Xiying Zhang, Mathias Neumann Andersen, Kirsten Kørup, Kiril Manevski

**Affiliations:** 1 Department of Agroecology, Aarhus University, Blichers Allé 20, 8830 Tjele, Denmark; 2 Sino-Danish College (SDC), University of Chinese Academy of Sciences, Eastern Yanqihu Campus, 380 Huaibeizhuang, Huairou, Beijing 101400, China; 3 Center for Agricultural Resources Research, Institute of Genetics and Developmental Biology, Chinese Academy of Sciences, Shijiazhuang 050021, China

**Keywords:** water deficit index, evapotranspiration, UAV, fraction of transpirable soil water, irrigation requirements

## Abstract

Agricultural droughts cause a great reduction in winter wheat productivity; therefore, timely and precise irrigation recommendations are needed to alleviate the impact. This study aims to assess drought stress in winter wheat with the use of an unmanned aerial system (UAS) with multispectral and thermal sensors. High-resolution Water Deficit Index (WDI) maps were derived to assess crop drought stress and evaluate winter wheat actual evapotranspiration rate (ET_a_). However, the estimation of WDI needs to be improved by using more appropriate vegetation indices as a proximate of the fraction of vegetation cover. The experiments involved six irrigation levels of winter wheat in the harvest years 2019 and 2020 at Luancheng, North China Plain on seasonal and diurnal timescales. Additionally, WDI derived from several vegetation indices (VIs) were compared: near-infrared-, red edge-, and RGB-based. The WDIs derived from different VIs were highly correlated with each other and had similar performances. The WDI had a consistently high correlation to stomatal conductance during the whole season (R^2^ between 0.63–0.99) and the correlation was the highest in the middle of the growing season. On the contrary, the correlation between WDI and leaf water potential increased as the season progressed with R^2^ up to 0.99. Additionally, WDI and ET_a_ had a strong connection to soil water status with R^2^ up to 0.93 to the fraction of transpirable soil water and 0.94 to the soil water change at 2 m depth at the hourly rate. The results indicated that WDI derived from multispectral and thermal sensors was a reliable factor in assessing the water status of the crop for irrigation scheduling.

## 1. Introduction

Agricultural drought—the lack of soil water for crop uptake—is the largest environmental stress significantly impacting wheat yield in all climates [1]. Crops stressed from high temperatures and a lack of water supply lose productivity and reduce growth duration [2]. At a global scale, irrigation increases the attainable yield of winter wheat by almost 35%, while in the North China Plain (NCP) yield benefits due to irrigation are even higher due to greater evaporative demands [3]. The latest developed technologies, such as unmanned aerial platforms equipped with multispectral and thermal sensors (UAS), allow efficient field monitoring for the early detection of drought with faster response and special attention to be paid to spatial heterogeneity and patterns within the field [4,5]. However, a deeper understanding of plant physiological responses to drought and their interpretation by the sensors is needed in order to further improve UASs for advanced precision agriculture use.

Winter wheat yield is highly susceptible to drought stress, especially at the flowering and grain-filling stage in particular if the drought events occur for prolonged periods [6]. Under such conditions, plants will decrease the number of grains per spike and reduce the grain weight, so the timely application of irrigation is necessary to prevent yield loss [6]. One of the early signals of winter wheat drought stress is the reduction in transpiration through reducing stomatal opening [7], which leads to an increase in the plant leaf temperature. Canopy temperature variations can be detected by thermal infrared sensors and can be used as evaluation criteria for plant water needs [8,9,10].

Thermal imagery is highly sensitive to environmental conditions and prone to distortions during acquisition and processing [11], and further interpretation may become more challenging due to the inability to separate canopy and soil in a frame [12]. These effects can be alleviated complementarily by visible/near-infrared images in order to evaluate crop canopy coverage since UAS multispectral imagery has a high correlation to leaf area index [13].

It is possible to accurately calculate the theoretical difference between canopy and air temperature for the canopy that fully transpires (no signals of drought stress) and the canopy that in theory fully stops the transpiration process (or experiences the maximum amount of drought stress). Moran et al. (1994) [14] combined this information by plotting it into a two-dimensional space of the canopy–air temperature difference and the canopy coverage into the Vegetation Index-Temperature (VIT) trapezoid and called it the Water Deficit Index (WDI). WDI is a close approximation of evapotranspiration as it shows the difference between fully transpiring and non-transpiring canopies; therefore, can be used to derive high-resolution evapotranspiration (ET) maps.

With the increased use of UAS equipped with multispectral and thermal cameras, it has become easier to spatially resolve the crop water status of an area by placing each pixel location in the VIT space, thus obtaining precise information about current crop conditions and water needs. WDI was successfully used to assess crop drought stress on various scales [15,16,17,18], mostly as a series of snapshots across the season, although the diurnal variation (during the day) may reveal more information about the crop stress symptoms [19]. Most studies use NDVI (Normalized Difference Vegetation Index) and SAVI (Soil-Adjusted Vegetation Index) as approximations of the fraction of vegetation cover in the WDI [16,17,18,20], but it is worth assessing the use of other multispectral vegetation indices (VIs) in WDI calculations, as other indices may provide more information on canopy status [21] and therefore improve calculations of WDI. One way to obtain a better approximation of the fraction of vegetation cover is to include red-edge-based vegetation indices that have a bigger advantage in capturing canopy structure compared to NIR-based indices [21]. Improved WDI calculations will provide a better baseline for the ET calculations and improve crop water need approximation.

The main objectives of this study were as follows:

- Improve and evaluate WDI derivation for winter wheat crop grown under a large variation of soil water conditions over the growing season using several multispectral indices, with specific attention to the seasonal variation and the diurnal changes in winter wheat growth.
- Establish and assess the relationship between WDI drought maps with field-measured parameters, such as stomatal conductance, leaf water potential, and actual soil water content.
- Design a framework for deriving high-resolution ET_a_ maps using a dual crop coefficient ET calculation combined with the WDI approach and evaluate the performance of ET calculations by validation against soil water balance.

## 2. Materials and Methods

### 2.1. Field Experimental Setup

**Figure 1:**
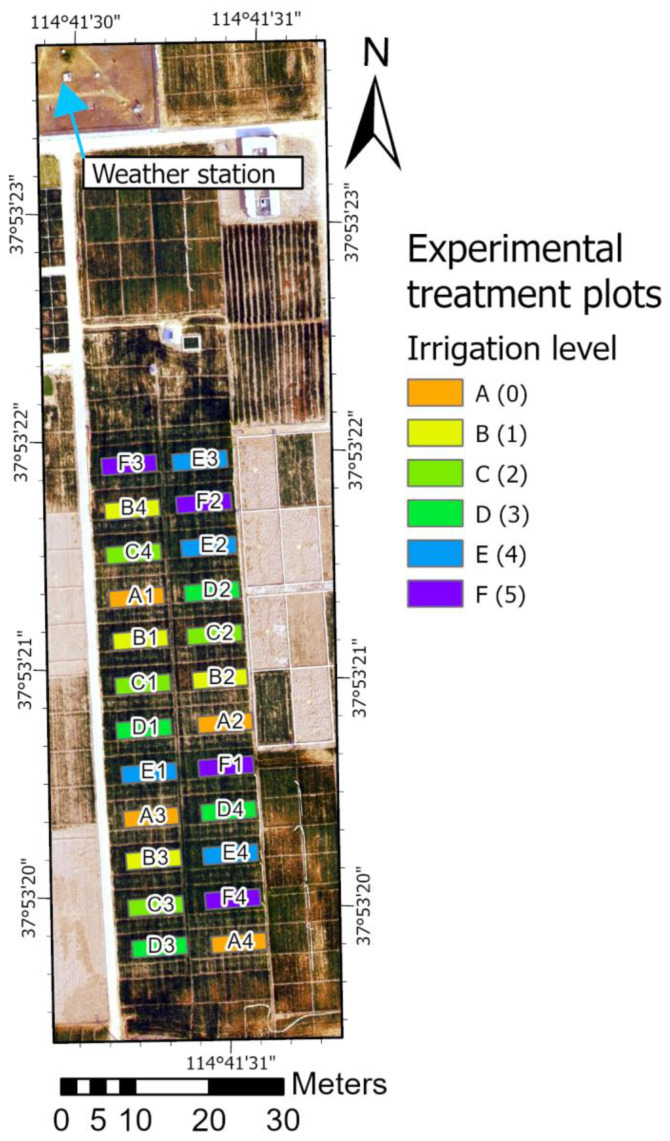
Outline of the experimental field setup at Luancheng Agro-Ecological Experimental Station with the location of the weather station and layout of the experimental treatment plots (codes are shown in Table 1).

Field experiment with winter wheat (*Triticum aestivum* L., variety Shixin633) was conducted at Luancheng Agro-Ecological Experimental Station (37°53′15″ N, 114°40′47″ E, elevation 50 m), located in the northern part of NCP. The climate is temperate semi-humid and monsoon-influenced. The main cropping system in the NCP is winter wheat and maize, an annual double cropping system with the straws from both crops being incorporated back into the soil system. The soil is silty loam, representative of the predominant soils located in the region. Figure 1 shows the layout of the six irrigation treatments at Luancheng station. The treatments included no irrigation during the growing season (rainfed) and incrementally added one to five irrigations. Each treatment had four replicates, summing a total of 24 plots, each of 4 × 9 m^2^ size with a 2 m zone between to minimize the mutual effects. Irrigation was performed by pumping water from a nearby well and the groundwater was transported to each plot by a low-pressure pipe. The irrigation amount was recorded by a flow meter, and for each irrigation, around 60–80 mm of water was applied. Table 1 lists the irrigation timing for different treatments during the two seasons.

The winter wheat was sown on 8 October 2018 and 14 October 2019 with a seeding rate of 187.49 kg/ha. Before sowing, 900 kg/ha compound fertilizer (N:P_2_O_5_:K_2_O = 19%:21%:5%) was incorporated into the top soil with cultivation. At the jointing stage, urea of 225 kg/ha (46% N) was applied with the irrigation in March or with a rainfall event for the rain-fed treatment. Winter wheat was managed free of weeds and diseases.

A weather station located 50 m from the experimental field automatically collected hourly measurements of solar radiation, air temperature, relative humidity, wind speed, precipitation, and ground heat flux measured by sensor at 5 cm depth below the soil surface above living grass. Volumetric soil water content was measured with neutron probe (503 DR, CPN International Inc., Concord, CA, USA) for each 20 cm soil layer until 2 m depth for 12 plots (2 per treatment). The fraction of transpirable soil water (FTSW) was calculated from the measurements by dividing available soil water by total available water (difference between field capacity and wilting point). Additionally, automatic soil water sensors (Insentek, Eastern Ecology Company, Beijing, China) for collecting hourly soil volumetric water content by 10 cm increment for the top 2 m soil profile were installed on 4 plots (treatments A, B, C, and F) in 2019 and one for each treatment in 2020. The average field capacity for the soil at the experimental site was 36% (*v*/*v*) and the wilting point was 13% (*v*/*v*) for the 2 m soil profile.

Stomatal conductance (g_s_) was measured on three plants in one plot per treatment on the youngest fully developed leaf with a leaf porometer (Model SC-1, Decagon, Pullman, WA, USA) on the day of the drone flights. It was measured between 10:00 and 15:00 h in full sunlight. Leaf water potential was measured shortly after using a pressure chamber on the same leaves as stomatal conductance.

At maturity, around 9 m^2^ from the center of each plot was manually harvested and threshed to obtain the grains, followed by air-drying the grains to a constant weight (13% water contents) and weighing to obtain the grain dry matter yield.

### 2.2. Unmanned Aerial System Acquisition of Multispectral and Thermal Images

An unmanned aerial vehicle (UAV) with mounted either multispectral Parrot Sequoia 1.2 MP, 1280 × 960 px (bands: green at 550 nm ± 40 nm; red at 660 nm ± 40 nm; red edge at 735 nm ± 10 nm; near-infrared at 790 nm ± 40 nm) or thermal sensor DJI Zenmuse XT uncooled Vox Microbolometer 640 × 512 px was operated at a flight altitude of about 100 m over the experimental field, resulting in 10 cm spatial resolution images. The orthomosaics of multispectral and thermal images were processed in Pix4Dmapper software (Pix4D SA, Prilly, Switzerland) with the use of “Ag Multispectral” and “Thermal camera” modes, respectively. The output was high-resolution GeoTIFF radiometrically calibrated images. The calibration of the multispectral images was performed with the MicaSense calibration panel images obtained prior to the flight and used during the processing in the Pix4Dmapper software. Thermal orthomosaics were calibrated according to the hottest (soil) and the coolest (wet canopy) pixels in the image. The flights were performed over the spring season of 2019 with additional diurnal flights in spring 2020. The flight times and main atmospheric parameters, such as air temperature, solar radiation, wind speed, relative humidity, and reference evapotranspiration, are presented in Table 2.

### 2.3. Calculation of Multispectral and Thermal Indices and Evapotranspiration Estimation

#### 2.3.1. Vegetation Indices

**Figure 2:**
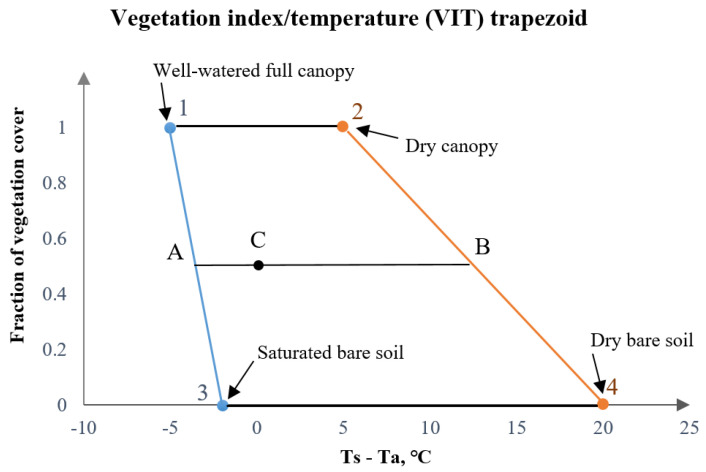
Illustration of the Vegetation Index/Temperature (VIT) trapezoid method. The *x*-axis shows surface–air temperature difference (Ts − Ta; °C) and the *y*-axis is a fraction of vegetation cover. Points 1, 2, 3, and 4 are theoretical extremes of the VIT trapezoid.

In the Vegetation Index/Temperature (VIT) trapezoid (Figure 2) it is essential to accurately estimate the fraction of vegetation cover in order to achieve high precision in the WDI calculations. Most articles used near-infrared based indices, such as NDVI (Normalized Difference Vegetation Index) and SAVI (Soil-Adjusted Vegetation Index) [5,14,15,16,20]. In this study, we investigated the use of several near-infrared-, red-edge-, and RGB-based indices in order to assess suitability for use in the WDI calculation. The selected vegetation indices, formulas, and references are provided in Table 3.

#### 2.3.2. Water Deficit Index (WDI) Calculations

Moran et al. (1994) [14] developed the WDI, based on the combination of thermal and multispectral vegetation indices. Crop canopy fraction cover approximated by the use of the vegetation index in conjunction with the surface–air temperature difference (Ts − Ta) is shaped into the Vegetation Index/Temperature (VIT) trapezoid limited by “wet” and “dry” baselines for both vegetated and bare soil surfaces (Figure 2).

In principle, the WDI is designed to represent the ability of the crop to transpire depending on the current environment and weather conditions and independently of the percentage of vegetation cover. WDI can be calculated as follows:

(7) $$\mathrm{WDI}=1-\frac{{\mathrm{ET}}_{a}}{{\mathrm{ET}}_{p}}=\frac{{\left(T_{S}-T_{a}\right)}_{\min}-{\left(T_{S}-T_{a}\right)}_{\mathrm{mes}}}{{\left(T_{S}-T_{a}\right)}_{\min}-{\left(T_{S}-T_{a}\right)}_{\max}}$$

where ET_a_ and ET_P_ are actual and potential evapotranspiration rates [mm h^−1^], respectively, T_S_ and T_a_ are surface and air temperature [°C], respectively, and subscript “min” is minimum, “max” is maximum, and “mes” is measured surface–air temperature differences.

For all pixels not fully covered by the canopy, WDI equals the ratio of distances AC/AB (Figure 2). In order to calculate the minimum and maximum theoretical values of (T_S_ − T_a_) depending on the fraction of vegetation cover, the baseline 1–3 in Figure 2 can be described by x = a *+* b*y and baseline 2–4 is x = c *+* d*y, where x is (T_S_ − T_a_), y is vegetation index VI, so Equation (7) becomes the following:

(8) $$\mathrm{WDI}=\frac{\left(a+\mathrm{bVI}\right)-{\left(T_{S}-T_{a}\right)}_{\mathrm{mes}}}{\left(c+\mathrm{dVI}\right)-\left(a+\mathrm{bVI}\right)}$$

Four theoretical cornets of the VIT trapezoid can be calculated by the following formula:

(9) $$\left(T_{s}-T_{a}\right)=\left[\frac{r_{a}\left(R_{n}-G\right)}{{\rho C}_{V}}\right]\left[\frac{\gamma \left(1+\frac{r_{c}}{r_{a}}\right)}{\left\{\Delta +\gamma \left(1+\frac{r_{c}}{r_{a}}\right)\right\}}\right]-\left[\frac{\mathrm{VPD}}{\left\{\Delta +\gamma \left(1+\frac{r_{c}}{r_{a}}\right)\right\}}\right]$$

where r_a_ is aerodynamic resistance [sm^−1^], R_n_ is net radiation [Wm^−2^], G is soil ground heat flux [Wm^−2^], C_v_ is the volumetric heat capacity of air (1200 J °C^−1^), ρ is air density [kg m^−3^], VPD is vapor pressure deficit [kPa], γ is the psychrometric constant [0.067 kPa °C^−1^], ∆ is the slope of saturation vapor pressure curve [kPa °C^−1^], and r_c_ is canopy resistance to vapor transport [s m^−1^]. Due to the differences in the conditions of each corner of the VIT trapezoid, r_a_, R_n_, G, and r_c_ were calculated separately according to procedures described by Antoniuk et al. (2021) [5]. The method accounts for the differences based on the vegetation cover as well as the differences between dry and wet bare soil.

#### 2.3.3. Actual Evapotranspiration (ET_a_) Calculations

Since WDI basically represents the ability of the crop to transpire, it may be possible to estimate crop evapotranspiration using WDI maps. As WDI is based on the dual approach (crop–soil), it is appropriate to use the dual-approach ET_a_ calculation proposed by Allen et al. (1998) [27] (Equation (10)):

(10) $${\mathrm{ET}}_{a}={\mathrm{ET}}_{0}\left(K_{\mathrm{cb}}K_{s}+K_{e}\right)$$

where K_cb_ is the basal crop coefficient, K_s_ is the drought stress coefficient, and K_e_ is the evaporation coefficient of the bare soil. The units of ET_a_ and ET_0_ are [mm h^−1^], and the latter was calculated by the Penman–Monteith equation [27].

In order to implement WDI into the ET calculation, we used it as a replacement for the drought stress coefficient (of the crop) and to correct the evaporation of soil depending on the fraction of vegetation cover (Equation (11)).

(11) $${\mathrm{ET}}_{a}={\mathrm{ET}}_{0}\left(K_{\mathrm{cb}}\left(1-\mathrm{WDI}\right)+0.25\left(1-f_{c}\right)\left(1-\mathrm{WDI}\right)\right)$$

More details about the calculation of all the specific parameters can be found in Moran et al. (1994) [14] and Antoniuk et al. (2021) [5]. The reference evapotranspiration rate was calculated using the Penman–Monteith equation [27]:

(12) $${\mathrm{ET}}_{0}=\frac{0.408\Delta \left(R_{n}-G\right)+\gamma \left(\frac{37}{T_{a\left(\mathrm{hr}\right)}+273}\right)u_{2}\left(e^{0}{}_{T_{a\left(\mathrm{hr}\right)}}-e_{a}\right)}{\Delta +\gamma \left(1+0.34u_{2}\right)}$$

where T_a(hr)_ is mean hourly air temperature [°C]; u_2_ is the wind speed at 2 m height [m s^−1^]; e^0^_Ta(hr)_ is the saturation vapor pressure at the temperature T_a_ [kPa]; e_a_ is actual vapor pressure [kPa]. R_n_ and G were calculated using the method described by Antoniuk et al. (2021) [5].

#### 2.3.4. WDI and ET_a_ Validation

In order to evaluate the performance of WDI and ET_a_ maps derived based on different multispectral indices, resulting maps were correlated to the field crop and soil parameters: stomatal conductance, leaf water potential, soil water contents, soil water change, and fraction of transpirable soil water (FTSW).

G_s_ and LWP values were averaged from the three measurements per treatment plots that were created as close to flight time as possible. For the correlations, the average WDI and ET_a_ values of all pixels in the plots per treatment were used (a total of 6 values).

For the correlation to the soil water contents and FTSW, average values per plot were used (a total of 24 values). For the plots that did not have the neutron probes installed, average values of measurements for each treatment were used. As for the soil water change, the data from automatic sensors were aggregated to daily values, the combined difference in the soil water level on the 2 m depth during the day was calculated for each hour based on the incoming radiation intensity, and the values (in mm/h) were correlated to the average WDI and ET_a_ values per treatment (total of 4 values in 2019 and 6 values in 2020).

In order to test the similarity between different WDIs, a two-tailed homoscedastic *t*-test was performed to determine whether there is a statistically significant difference between every two groups of WDI.

## 3. Results

### 3.1. Meteorological Conditions, Soil Water and Winter Wheat Physiological Variations for the Two Seasons

**Figure 3:**
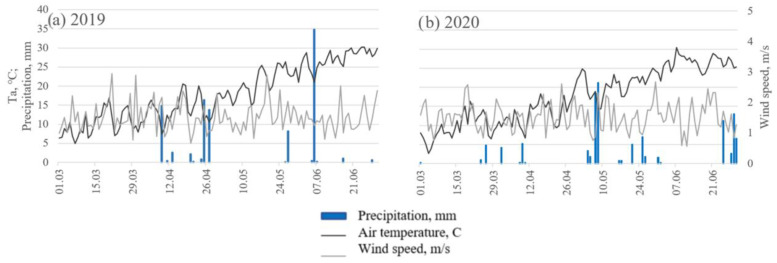
Maximum daily temperature, precipitation, and average wind speed for the spring season (March–June) of (**a**) 2019 and (**b**) 2020.

**Figure 4:**
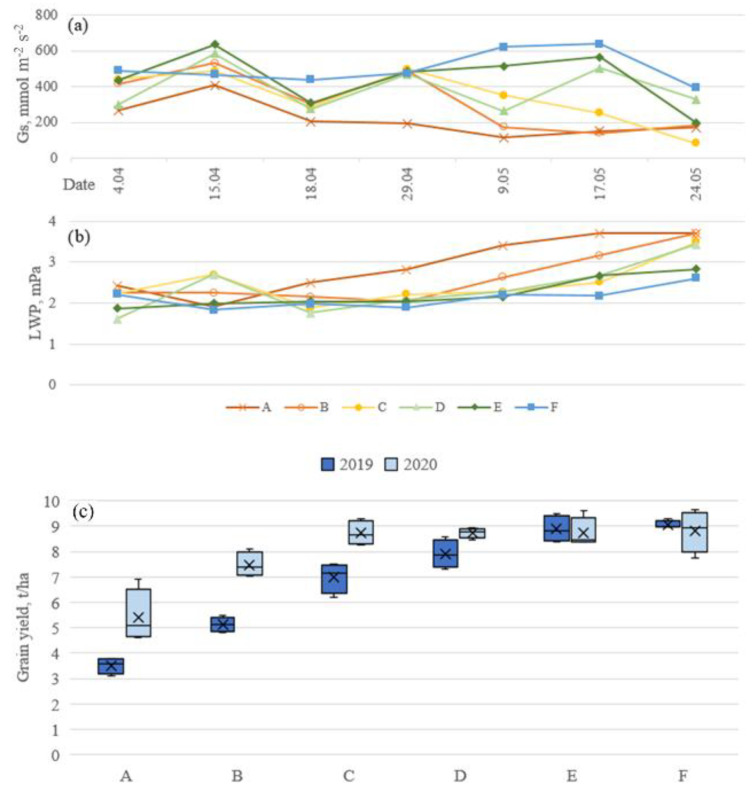
(**a**) Stomatal conductance (g_s_) for April–May 2019, (**b**) leaf water potential (LWP) for April–May 2019, and (**c**) grain yield in 2019 and 2020. The treatment names A–F refer to treatments from the lowest to the highest irrigation amounts, respectively.

The spring 2019 was characterized by much warmer and more stable weather, while in 2020 the temperature fluctuated more. There was a limited amount of rain in 2019 compared to 2020 and notably no rain during March 2019 (Figure 3). Due to this, the amount of water available for the winter wheat in 2019 was limited and the crop was mostly supplied by irrigation and as the season progressed, the physiological differences between treatments became more and more prominent (Figure 4). During the season, stomatal conductance was gradually decreasing, and in particular treatments with low irrigation reduced transpiration faster than the treatments with high irrigation (Figure 4a). On the 18th of April, stomatal conductance of all treatments was notably lower compared to other dates due to high wind conditions. Leaf water potential was gradually decreasing during the season until treatments A, B, and C reached LWP of −3.5 MPa because the leaves were drying out due to the maturity of the plant (Figure 4b).

In 2019, the amount of irrigation had a direct impact on the winter wheat grain yield (Figure 4c). Treatments with low irrigation (A, B, C) had significantly lower yield compared to treatments with higher amounts of irrigation (E, F). The yield of the non-irrigated treatment (A) was less than half of the treatments receiving sufficient irrigation in 2019. No significant difference was found between the two treatments with the highest irrigation amounts (E and F). In 2020, due to the higher amount of rainfall that was more evenly distributed through the season and slightly lower temperature, the yield was overall higher than in 2019 for the lower irrigation treatments, but treatments with higher irrigation amounts had similar yields as in 2019. In 2020, only treatments A and B had noticeably lower yields than the other treatments (Figure 4c). The yield difference in treatment A between 2019 and 2020 is attributed to the differences in the weather conditions.

### 3.2. WDI Maps Derived from the Different Multispectral Indices for the Entire Growing Season in 2019

**Figure 5:**
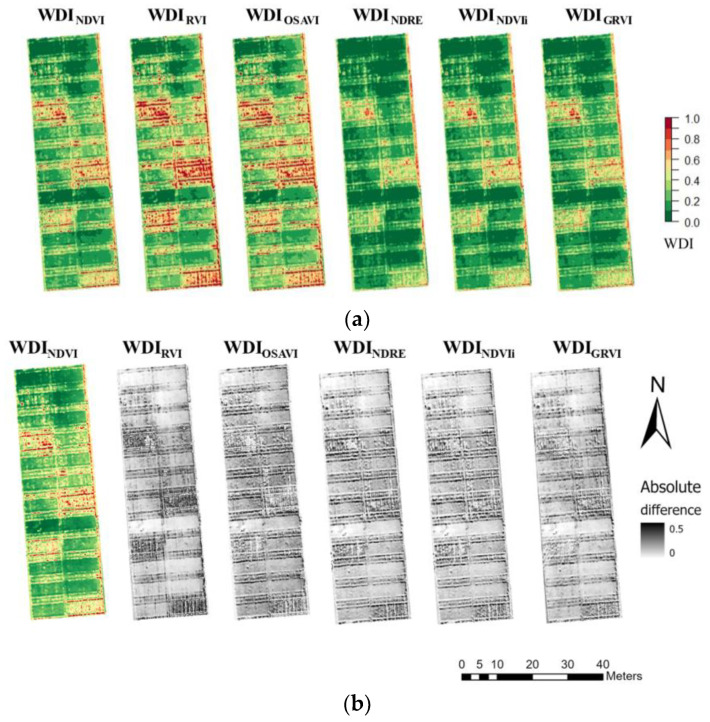
(**a**) Water Deficit Index (WDI) maps calculated from different multispectral indices for the winter wheat based on the flight carried out on 15 April 2019 and (**b**) absolute difference map between WDI_NDVI_ and other WDIs. NDVI is the Normalized Difference Vegetation Index; RVI—Ratio Vegetation Index; OSAVI—Optimized Soil-Adjusted Vegetation Index; NDRE—Normalized Difference Red Edge; NDVIi—Red and Red Edge NDVI; GRVI—Green and Red ratio Vegetation Index.

**Figure A1:**
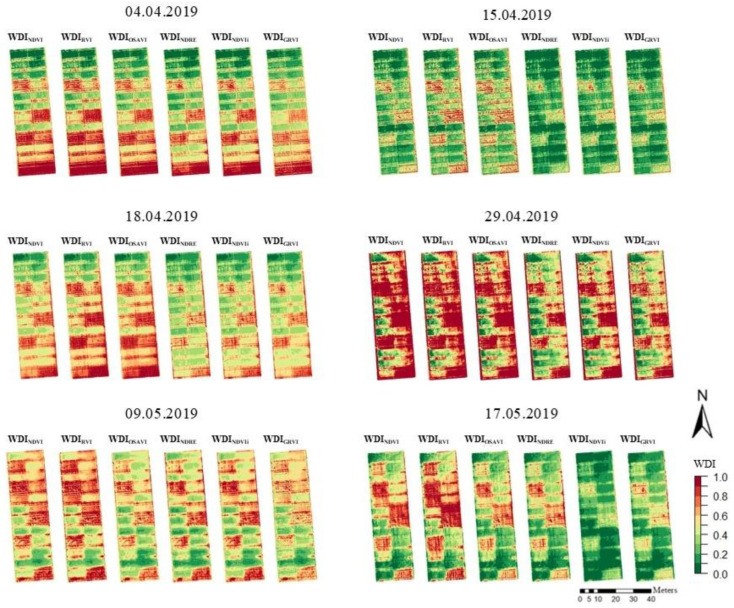
WDI maps calculated from different multispectral indices for the winter wheat growing season of 2019 (April–May).

High spatial resolution WDI maps were derived for the growing season, with a fraction of vegetation cover derived by different multispectral indices—NDVI, RVI, OSAVI, NDRE, NDVIi, and GRVI—referred to hereafter as WDI_NDVI_, WDI_RVI_, WDI_OSAVI_, WDI_NDRE_, WDI_NDVIi_, and WDI_GRVI_, respectively (Figure 5a and Appendix A Figure A1). Figure 5b shows the absolute difference maps between WDI_NDVI_ and the other WDIs on 15 April 2019. WDI_RVI_ had consistently higher values compared to the others. All WDI indices were highly correlated with each other (Table 4). By performing a two-tailed homoscedastic *t*-test, it was found that WDI_NDVI_, WDI_RVI_, and WDI_OSAVI_ did not differ statistically from each other. Similarly, WDI_NDRE_, WDI_NDVIi_, and WDI_GRVI_ did not have differences statistically between each other. However, WDIs from the two groups were statistically different from each other. On the thermal image from 4 April 2019 there was thermal drift observed, so these data were removed from further analysis.

### 3.3. Correlations of WDI to Winter Wheat Physiological Parameters (Stomatal Conductance, Leaf Water Potential) and Yield in 2019

Table 5 shows correlations of WDI based on different VI to the g_s_, LWP, and winter wheat yield for the season of 2019.

WDI was consistently highly correlated to stomatal conductance. The exception was the 18 April, likely due to effects of higher wind and, the 15 April due to the earlier stage of winter wheat development. At the end of the season, WDI_RVI_ had a higher correlation to stomatal conductance compared to other vegetation indices, but there was no apparent difference earlier in the season. With respect to leaf water potential, correlation to WDI for all VI was getting higher with the growth of the crop and the difference in soil water conditions becoming larger. This can be explained by the fact that at the beginning of the season, there was a very small variation in stomatal conductance and leaf water potential among the six treatments (Figure 4a). As for the correlation to the yield, all indices performed similarly having higher correlation by the end of the season (R^2^ up to 0.91) compared to the middle of the season (R^2^ = 0.5; Table 5), although red-edge-based indices had slightly better correlation.

### 3.4. Diurnal Variation of WDI and Its Correlation to Winter Wheat Physiological Parameters and Soil Water Status in 2020

**Figure 6:**
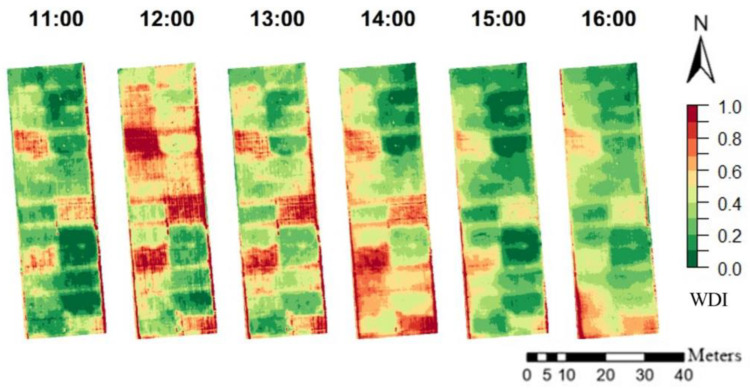
WDI maps calculated based on NDVI for the diurnal winter wheat flights on 23 April 2020.

**Figure A2:**
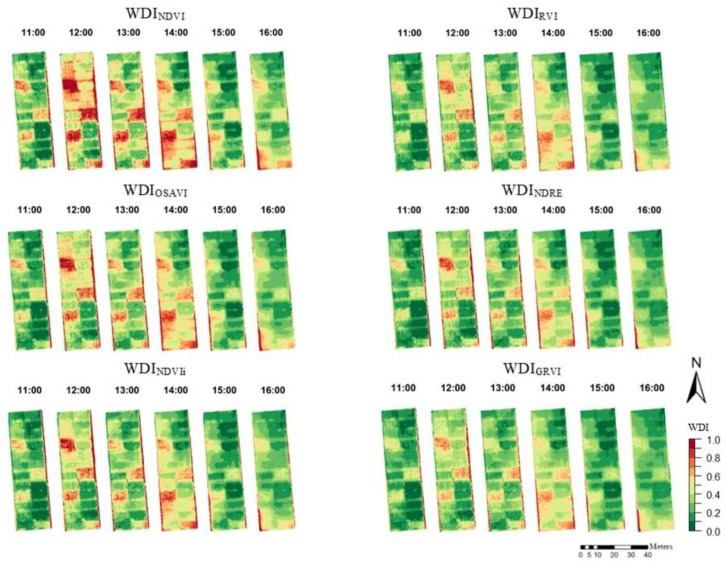
WDI maps calculated from different multispectral indices for the diurnal winter wheat flights on 23 April 2020.

WDI maps of the diurnal flights on 23 April 2020 are presented in Figure 6 and Appendix A Figure A2. Correlations to SWC (mm), g_s_ (mmol m^−2^ s^−2^), and LWP (mPa) are represented in Table 6. During the day, WDI increased from 11:00 to 12:00, remained high for the treatments with low irrigation, and decreased for all treatments after 14:00. The WDI correlations to the soil water content, stomatal conductance and leaf water potential were high throughout the whole day and are presented in Table 6. Correlations to the SWC were consistently high and negative during the whole day (R^2^ between −0.96 and −0.99) and with all the VIs except for the flight at 16:00 when R^2^ was between −0.88 and −0.89, possibly due to lower variation between the treatments. In general, WDIs at 14:00 had the same or higher correlations to winter wheat g_s_ and LWP compared to other hours, although those measurements were performed around 10–11.

### 3.5. Seasonal and Diurnal ET_a_ Derived from Different WDIs and Their Connection to Soil Water Status

**Figure 7:**
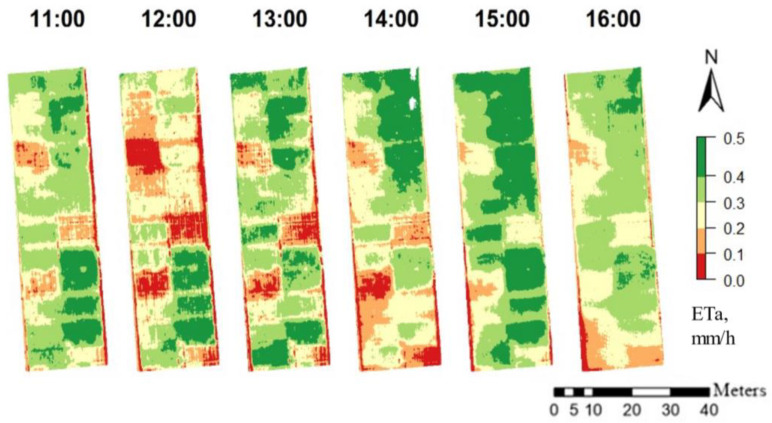
ET_a_ maps of the diurnal flights on 23 April 2020 calculated based on WDI_NDVI_.

**Figure 8:**
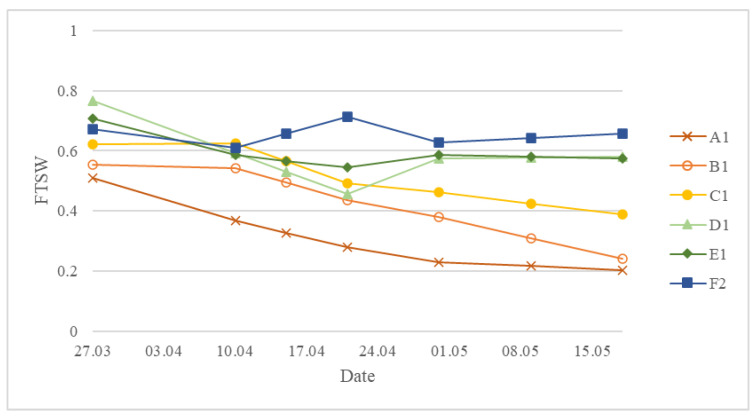
Seasonal change of fraction of transpirable soil water (FTSW) in March–May 2019 calculated from the volumetric soil water content measured with the neutron probe. The treatment names A–F refer to treatments from the lowest to the highest irrigation amounts, respectively, with the numbers next to the letter corresponding to the replicate number.

**Figure 9:**
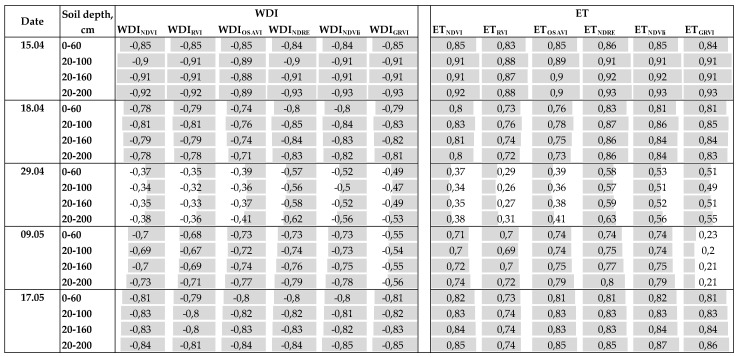
WDI and ET correlations to fraction of transpirable soil water (FTSW) at various root depths for the April–May of 2019. Grey color in the cells visually represents the correlation value.

ET_a_ maps calculated based on WDI_NDVI_ of the diurnal flights on 23 April 2020 are presented in Figure 7. The seasonal change of FTSW in 2019 is provided in Figure 8. The WDI correlation to FTSW was consistently high during the season of 2019 (Figure 9). The exception was 29 of April because two days prior there was rain, so the soil had enough water but the crop treatments with lower irrigation still showed signs of stress by having lower g_s_ and higher LWP (Figure 4a,b). This points to difficulties for the prediction of soil water using remote sensing if the crop shows signs of stress even after the topsoil water has been replenished. Similarly to WDI, actual evapotranspiration ET_a_ maps that were calculated based on WDIs had very similar correlations to FTSW. ET_GRVI_ had the worst performance overall.

**Figure 10:**
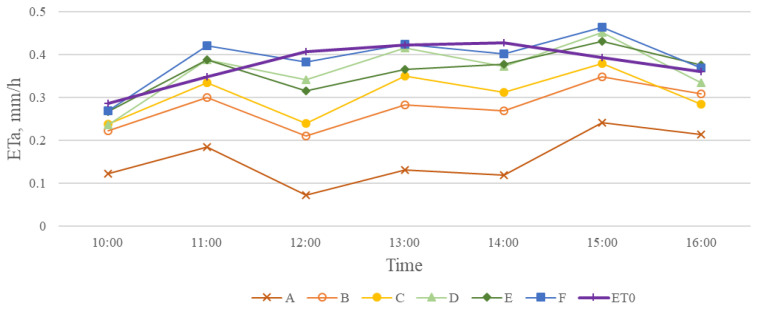
ET_aNDVI_ (mm h^−1^) derived from WDI_NDVI_ diurnal variation on 23 April 2020. The treatment names A–F refer to treatments from the lowest to the highest irrigation amounts, respectively. Reference evapotranspiration ET_0_ (mm h^−1^) was calculated using the Penman–Monteith equation [27].

**Figure 11:**
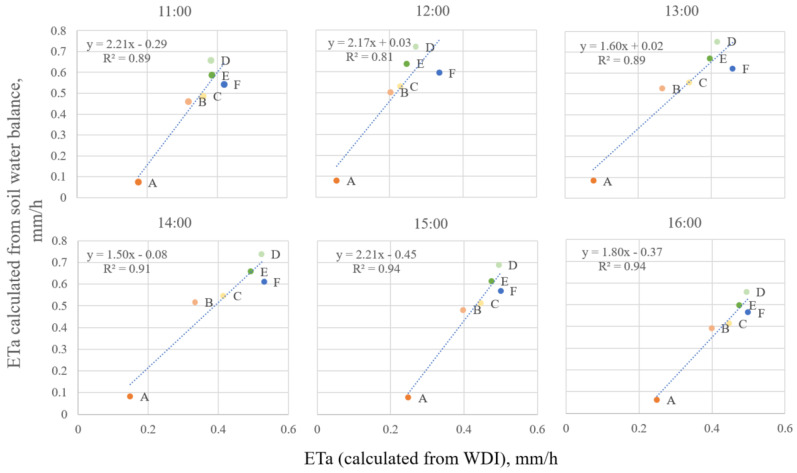
Correlation between ET_a_ (mm h^−1^) calculated from WDI_NDVI_ and ET_a_ (mm h^−1^) calculated from soil water balance during the day of 23 April 2020. The treatment names A–F refer to treatments from the lowest to the highest irrigation amounts, respectively.

On the diurnal scale, ET_a_ varied during the day (Figure 10). The lowest ET was observed at 10:00 and the highest was at 15:00 after the noon maximum air temperature passed. ET_a_ was highly correlated to the change in the soil water to 2 m depth (Figure 11). The change in the soil water from 11:00 to 15:00 was generally higher than winter wheat potential evapotranspiration; therefore, it may be assumed that part of the soil water was lost due to the water movement to the deeper soil layers.

## 4. Discussion

### 4.1. Difference between Vegetation Indices in the Calculation of WDI

WDI combines thermal and multispectral remotely sensed data, and the method relies on good delineation of crop coverage (*x*-axis in Figure 2). LAI approximation using various VIs based on combinations of RGB and near-infrared spectral bands has shown good results [13,21]. However, it is known that near-infrared (e.g., NDVI) tends to saturate at high canopy densities [21], so it may be beneficial to make use of red-edge spectral bands (e.g., NDRE and NDVIi, Table 3, Equations (4) and (5)). On the other hand, multispectral sensors tend to be more expensive, so substituting near-infrared- and red-edge-based VIs with RGB-based VIs may be more beneficial for the user [28].

In this study, we investigate which VI performs better in WDI calculations by comparing WDIs to winter wheat drought stress indicators—g_s_ and LWP—as well as soil water status. The results show little difference between the performances of different indices (Table 4). Near-infrared-based indices were very closely related to each other, and red-edge-based indices performed similarly to near-infrared-based indices. As expected, WDI based on RGB (WDI_GRVI_) performed slightly worse compared to near-infrared-based, which can easily be explained by the absence of information about the canopy structure that is mostly present in the near-infrared region [13]. However, in the absence of multispectral sensors, it is feasible to derive high-resolution WDI maps using GRVI as a fraction of vegetation cover approximation and still have a reliable relation to winter wheat water status.

### 4.2. WDI Connection to Winter Wheat Physiological Parameters

On the seasonal scale in 2019, we achieved good relations to both g_s_ and LWP due to the high variability between the treatments that were especially prominent by the end of the season. The stomatal behavior of winter wheat is very susceptible to environmental fluctuation [29]. The lowest correlation was observed on 15 April (R^2^ = −0.61–−0.65, Table 5) and 18 April (R^2^ = −0.78–−0.79, Table 5) and the highest on 29 April (R^2^ = −0.98–−0.99, Table 5) in the 2019 season. The high correlation on 29 April may be explained by the flight time (12:00) when there was the most prominent drought on the WDI map.

WDI was not able to accurately outline the differences in LWP between the treatments at the beginning of the season (Table 5) due to the low variation in LWP in the crop’s early development, e.g., the difference between high and low irrigation experimental fields on 15 April was much less than on 17 May. Additionally, the irrigation amounts on 15 April were similar among treatments C, D, E, and F. Differences in LWP by the end of the season were caused by drought-stressed plants that started to mature earlier compared to the treatments that had enough water supply. Drought stress shortens winter wheat growing seasons and results in lower yields [2], and flowering and grain-filling stages are the most susceptible, especially when extreme drought events occur [6].

The yield difference between treatments A and B in 2019 and 2020 (Figure 4) is attributed to the weather conditions in March (Figure 3). In 2019, there was no rain and the air temperature increased more rapidly than in 2020. As treatment A is completely rainfed, the impact of prolonged drought was severe. These differences are reflected in WDI maps as WDI in 2020 for treatment A shows lower values than that in 2019 for a similar period (Figure 5 and Figure 6, Appendix A Figure A1 and Figure A2). The high difference in yield in low irrigation treatments between the years 2019 and 2020 highlights the importance of sufficient irrigation during the early development stages of winter wheat. This may be supported by the relatively high correlation of WDI to yield in 2019 even from the early flights in the season (R^2^ up to −0.86 on 15.04, Table 5). As expected, the highest correlation to the yield was observed on the last flight on 17 May which was closer to harvesting time (Table 5). For most of the measurement days, WDI based on RE indices (NDRE and NDVIi) had stronger correlations compared to others.

Winter wheat yield did not exceed 10 t/ha (treatments E and F in 2019 and C, D, E, and F in 2020, Figure 4) even with an increased amount of irrigation, which points to the necessity to create new drought-resilient breeds in order to increase yields even more under the same amounts of water use. As the screening for new varieties and appropriate phenotyping require a significant amount of time and resources, it is worth investigating the use of WDI for the variety screening for the new drought-resistant breeds in future research.

Most of the thermal studies focus on single snapshots as an observation of drought stress [30,31,32], and there is a possibility for incorrect representation of the actual water status of the crop. The environmental conditions greatly fluctuate during the day, and this impacts the canopy temperature and the baseline calculations, e.g., high/low wind conditions may lead to under—or overestimating aerodynamic resistance [33]. Additionally, crops have different potential evapotranspiration during the day depending on the light intensity. In this research, we conducted several flights during the day on 23 April 2020 with the interval of 1 h from 11:00 to 16:00 in order to study the diurnal fluctuations of canopy temperature and consequently winter wheat drought stress. The WDI was not stable during the day (Figure 6). The WDI increased until 12:00 and then gradually declined until 15:00 with a slight increase at 16:00. Although g_s_ and LWP were measured between 10:00 and 12:00, there was a consistently high correlation to those variables from all the flights (Table 6) with R^2^ between 0.93 and 0.96 for LWP and R^2^ between −0.82 and −0.89 for g_s_.

### 4.3. WDIs Use in ET Calculation and Its Connection to the Soil Water Variation

WDI has the potential to predict soil water content based on the crop water status. It should be noted that even after rain or irrigation the crop shows signs of drought stress (e.g., 29 April 2019, Appendix A Figure A1). This will lead to higher WDI values and will be interpreted as depletion in soil water. Therefore, WDI prediction of the soil water content should be used with caution and evaluations should consider the prior weather conditions and irrigation events. Accordingly, this applies to the evapotranspiration that was calculated based on WDI.

In this study, we chose the FAO dual approach in order to account for the soil background during the calculation [27] (Equation (11)). Both during the season as well as diurnally, ET_a_ maps were closely following WDI patterns—lower WDI values represented areas with higher transpiration rate and ET_a_ was close to the potential level and high WDI areas translated to the areas with low ET_a_ (Figure 7).

In this study, we tested the correlation of WDI and ET_a_ to the fraction of soil transpirable soil water on different soil depths. The results show that a higher correlation was achieved when we considered soil depth from 10 to 200 cm (Figure 9). This showed the water in the subsoil still had high impact on crop water status, which was also observed in the study of Wang et al. (2018) [34]. Additionally, this demonstrated that roots were likely present up to 200 cm depth, in accordance with Zhang et al. (2004) [35], and that these could efficiently extract the water to supply plant demands. The approach opens a possibility to use WDI to find the rooting depth of winter wheat so that WDI may be used in high-throughput phenotyping as a screening tool and for the selection and delineation of varieties with superior rooting depth and root distribution [36,37], and its application in the selection of drought-resistant varieties. The current study showed that subsoil moisture is important to plant water supply and that it is essential to consider soil water content for the entire root zone and not only the topsoil.

On the diurnal scale on 23 April 2020, the variation in ET_a_ may be attributed to the amount of incoming radiation and change in air temperature and other atmospheric parameters (Figure 10). High ET_a_ correlation to the ET_a_ calculated using a change in the soil water to 2 m depth (Figure 11) implies the relation of thermal imagery to the in-field water balance. However, the change in the soil water from 11:00 to 15:00 was higher than winter wheat potential ET; therefore, it was assumed that part of the soil water was lost due to water movement to the deeper soil levels, i.e., concurrent percolation. The correlation in the afternoon hours was higher than the noon with R^2^ = 0.94 at 15:00 and 16:00 (Figure 11). This implies that the assessment of ET_a_ may be more reliable after the noon heat wave has passed and the crop canopy has cooled down (Figure 7), due to an increase in wind speed and a decrease in solar radiation (Table 2).

### 4.4. Quality Control of Thermal Data and Atmospheric Conditions Impact the WDI Derivation

In the case of WDI, the surface temperature plays a much more important role than the accuracy of the fraction of vegetation cover estimation. Thermal images on 4 April 2019 and 10 am 23 April 2020 had thermal drift, so the part of the field appeared to be hotter than the other part due to the heating of the camera during the flight. It is difficult to correct this type of temperature drift in the calibration process, and as a result the maps were discarded from the analyses.

There is a big impact of wind on WDI performance. As can be seen from the results on the 18 April, the presence of high wind greatly decreased the performance of WDI to represent g_s_ and LWP compared to the 29 April (Table 5). At a higher wind speed, there is a tendency to underestimate aerodynamic resistance [38], which will lead to a wrong interpretation of the drought response in the crop. High wind speed conditions could have been a cause of thermal drift at 10 am on 23 April 2020, as the average wind speed at that hour was higher than the wind speed later in the day. Similarly, very low wind may lead to an overestimation of the aerodynamic resistance [38], which will lead to an overestimation of the WDI values. As an alternative, it is possible to investigate wind-speed-independent models for determining crop evapotranspiration on a higher scale [33].

Other considerations for the quality of thermal image output and the resulting WDI include viewing geometry, as the signals from nearby areas may influence the studied area [39], and flight height [40], which was around 100 m in this study.

## 5. Conclusions

In this study, we derived high-resolution winter wheat WDI and ET maps by the means of multispectral and thermal imagery acquired with UAS in combination with the energy balance model. The resulting maps were compared to the field measurements of soil water and crop stomatal conductance and leaf water potential. Following the aims of the study, the results can be summarized as follows:

- High-resolution WDI maps were derived over the winter wheat growing season in north China using several multispectral indices, and we determined that different VIs—near-infrared, red-edge and RGB methods—were closely related to each other and had only a small influence on the WDI results.
- The study established and evaluated the relationship between WDI drought maps with field-measured parameters, such as g_s_, LWP, and soil water status. WDI based on the red edge had better relation to LWP, WDI based on near-infrared had a stronger correlation to g_s_, and WDI based on RGB had an overall worse performance.
- High-resolution ET_a_ maps could be derived using a dual crop coefficient ET calculation combined with the WDI approach. ET_a_ was highly correlated to both crop and soil water status variables, such as g_s_, LWP, soil water content, FTSW, and soil water change to 2 m depth.

## Figures and Tables

**Table 1:** Timing of each irrigation to different treatments for the season of 2019 and 2020 (60–80 mm per irrigation).

Treatment Abbreviations (Irrigation Numbers)	2018	2019					2020			
	Season 2019					Season 2020				
A (0)	No irrigation					No irrigation				
B (1)		29.03					25.03			
C (2)		29.03	05.05				25.03	01.05		
D (3)		15.03	26.04	16.05			17.03	21.04	14.05	
E (4)	30.11	29.03	26.04	16.05		28.11	25.03	29.04	19.05	
F (5)	30.11	29.03	19.04	05.05	16.05	28.11	25.03	13.04	01.05	19.05

**Table 2:** Flight dates, time, and main environmental parameters at the time of the flight that were used in the WDI calculations: air temperature T_a_ (°C), solar radiation Rs (Wm^−2^), wind speed u (m s^−1^), relative humidity RH (%), Penman–Monteith reference evapotranspiration ET_0_ (mm h^−1^).

Flight Date	Flight Time	Air Temperature Ta (°C)	Solar Radiation R_s_ (Wm^−2^)	Wind Speed u (m s^−1^)	Relative Humidity RH (%)	ET_0_ (mm h^−1^)
2019.04.04	11:00	17.5	731.5	2.4	51	0.41
2019.04.15	11:00	17.2	832.7	3.6	56	0.45
2019.04.18	11:00	20.5	671.8	5.9	39	0.38
2019.04.29	12:00	18.1	800.1	4.9	85	0.53
2019.05.09	10:00	22.9	626.9	1.9	39	0.39
2019.05.17	10:00	25.4	644.9	4.1	88	0.58
2020.04.23	11:00	14.6	840	4.6	30	0.35
	12:00	15.4	919.2	3.3	30	0.41
	13:00	16.2	961	3.9	30	0.42
	14:00	17.6	944.9	4.4	30	0.43
	15:00	17.9	881.2	5.6	30	0.39

**Table 3:** The selection of vegetation indices used for the calculation of Water Deficit Index (WDI) *.

Index	Description	Formula		References
NDVI	Normalized Difference Vegetation Index	$\mathrm{NDVI}=\frac{\mathrm{NIR}-R}{\mathrm{NIR}+R}$	(1)	Rouse J.W. et al. (1974) [22]
RVI	Ratio Vegetation Index	$\mathrm{RVI}=\frac{\mathrm{NIR}}{R}$	(2)	Pearson and Miller (1972) [23]
OSAVI	Optimized Soil-Adjusted Vegetation Index	$\mathrm{OSAVI}=\left(1+0.16\right)\frac{\mathrm{NIR}-R}{\mathrm{NIR}+R+0.16}$	(3)	Rondeaux et al. (1996) [24]
NDRE	Normalized Difference RedEdge	$\mathrm{NDRE}=\frac{\mathrm{NIR}-\mathrm{RE}}{\mathrm{NIR}+\mathrm{RE}}$	(4)	Gitelson and Merzlyak (1994) [25]
NDVIi	Red and RedEdge NDVI	$\mathrm{NDVIi}=\frac{\mathrm{NIR}-\left(0.4*R+0.6*\mathrm{RE}\right)}{\mathrm{NIR}+\left(0.4*R+0.6*\mathrm{RE}\right)}$	(5)	Xie et al. (2018) [21]
GRVI	Green and Red ratio Vegetation Index	$\mathrm{GRVI}=\frac{G-R}{G+R}$	(6)	Tucker (1979) [26]

*: Where G is green, R is red, RE is red edge and NIR is near-infrared band.

**Table 4:** Correlations between WDIs calculated from different multispectral indices calculated based on the whole season of 2019.

	WDI_NDVI_	WDI_RVI_	WDI_OSAVI_	WDI_NDRI_	WDI_NDVIi_	WDI_GRVI_
WDI_NDVI_	1					
WDI_RVI_	0.98	1				
WDI_OSAVI_	0.96	0.95	1			
WDI_NDRI_	0.96	0.95	0.94	1		
WDI_NDVIi_	0.92	0.87	0.92	0.92	1	
WDI_GRVI_	0.95	0.92	0.94	0.95	0.97	1

**Table 5:** WDI correlation to stomatal conductance, leaf water potential, and yield based on WDIs derived from different multispectral indices for the April–May 2019.

Date	WDI_NDVI_	WDI_RVI_	WDI_OSAVI_	WDI_NDRE_	WDI_NDVIi_	WDI_GRVI_
	Stomatal conductance (gs)					
15.04	−0.63	−0.63	−0.61	−0.65	−0.65	−0.64
18.04	−0.79	−0.79	−0.79	−0.78	−0.78	−0.78
29.04	−0.98	−0.98	−0.99	−0.99	−0.99	−0.99
09.05	−0.89	−0.90	−0.86	−0.81	−0.84	−0.90
17.05	−0.87	−0.90	−0.84	−0.83	−0.79	−0.82
	Leaf water potential (LWP)					
15.04	−0.06	−0.05	0.02	−0.14	−0.12	−0.12
18.04	0.55	0.54	0.47	0.63	0.61	0.60
29.04	0.92	0.90	0.91	0.94	0.94	0.91
09.05	0.94	0.91	0.96	0.99	0.98	0.85
17.05	0.96	0.93	0.96	0.96	0.95	0.96
	Yield					
15.04	−0.87	−0.87	−0.86	−0.87	−0.87	−0.86
18.04	−0.64	−0.64	−0.56	−0.70	−0.69	−0.67
29.04	−0.52	−0.50	−0.53	−0.69	−0.65	−0.61
09.05	−0.85	−0.83	−0.88	−0.89	−0.88	−0.70
17.05	−0.89	−0.84	−0.89	−0.90	−0.91	−0.91

**Table 6:** Correlation of average WDI for each irrigation treatment to soil water content (SWC, mm), stomatal conductance g_s_ (mmol m^−2^ s^−2^), and leaf water potential (LWP, mPa) (see Table 1 for treatment details). WDI is based on diurnal flights on 23 April 2020 and different multispectral indices.

WDI	Parameter	Hour					
		11:00	12:00	13:00	14:00	15:00	16:00
WDI_NDVI_	SWC	−0.98	−0.98	−0.98	−0.97	−0.98	−0.88
	g_s_	−0.87	−0.82	−0.85	−0.87	−0.82	−0.82
	LWP	0.96	0.93	0.93	0.97	0.95	0.96
WDI_RVI_	SWC	−0.98	−0.98	−0.98	−0.97	−0.98	−0.87
	g_s_	−0.85	−0.81	−0.84	−0.86	−0.81	−0.80
	LWP	0.95	0.92	0.91	0.96	0.94	0.95
WDI_OSAVI_	SWC	−0.98	−0.98	−0.98	−0.97	−0.98	−0.88
	g_s_	−0.87	−0.83	−0.85	−0.87	−0.82	−0.82
	LWP	0.96	0.94	0.93	0.97	0.94	0.96
WDI_NDRE_	SWC	−0.98	−0.98	−0.98	−0.97	−0.98	−0.89
	g_s_	−0.86	−0.82	−0.85	−0.86	−0.82	−0.82
	LWP	0.96	0.93	0.93	0.97	0.94	0.96
WDI_NDVIi_	SWC	−0.98	−0.98	−0.98	−0.97	−0.98	−0.89
	g_s_	−0.87	−0.83	−0.85	−0.87	−0.83	−0.83
	LWP	0.96	0.94	0.93	0.97	0.95	0.96
WDI_GRVI_	SWC	−0.98	−0.99	−0.97	−0.96	−0.98	−0.88
	Gs	−0.87	−0.85	−0.86	−0.89	−0.83	−0.83
	LWP	0.96	0.94	0.93	0.98	0.95	0.97

## Data Availability

The data presented in this study are available on request from the corresponding author.
